# Determining nutrients degradation kinetics of chickpea (*Cicer arietinum*) straw using nylon bag technique in sheep 

**Published:** 2012-05-17

**Authors:** A. Aghajanzadeh-Golshani, N. Maheri-Sis, A. Baradaran-Hasanzadeh, A. Asadi-Dizaji, A. Mirzaei-Aghsaghali, J. Dolgari-Sharaf

**Affiliations:** *Department of Animal Science, Shabestar Branch, Islamic Azad University, Shabestar, Iran*

**Keywords:** Chemical composition, Chickpea straw, Degradation, Nylon bag

## Abstract

Straw a by-product from grain legume crops is produced in large quantities in Iran. Straw is constant component of ruminant diets on small holder farms; however, there is little information about its nutritive value. Accordingly experiment was conducted to determine the chemical composition and ruminal organic matter (OM) and crude protein (CP) degradability of chickpea straw using nylon bags (*in situ*) technique. Replicated samples were incubated at 0, 2, 4, 8, 12, 24, 48 and 72 hours in three rumen canulated Ghezel rams with 50±3 kg body weight. Dry matter (DM), CP, ether extract (EE), OM, crude fiber (CF) and nitrogen free extract (NFE) content of chickpea straws were 92.2, 6.1, 5.5, 92.0, 34.3 and 46.2%, respectively. The soluble fraction (*a*) of the OM and CP of chickpea straw was 17.5 and 40.8% and potential degradability (*a+b*) of OM and CP was 56.7 and 72.0%, respectively. Effective degradability at different passage rates (2, 5 and 8% per hours) for OM was 51.0 44.9 and 40.7% and for CP were 68.4, 64.3 and 61.3%, respectively. In conclusion, based on chemical composition and degradation characteristics, chickpea straw could have moderate nutritive value for ruminants.

## Introduction

Cereals and legumes are cultivated to obtain grain for human consumption or for animal feed (Lopez *et al.*, 2005). Legume grains are important in meeting human dietary requirements in developing countries (Ramalho Ribeiro and Portugal Melo, 1990; Maheri-Sis *et al.*, 2008).

Among legume grains, chickpea (*Cicer arietinum*) is a widely grown crop and ranks first in grain legumes cultivated in Iran (Maheri-Sis *et al.*, 2007; Parsa and Bagheri, 2007). Chickpea production in Iran was 0.3 million ton with an area of 0.75 million ha (Parsa and Bagheri, 2007).

Crop residues after harvesting can produce substantial amount of biomass, often considered an agricultural waste. Straw is one of the main by-products from cereal and grain legume crops (Lopez *et al.*, 2005). After chickpea grain threshing, large amounts of straw (about 400 kg per ha) usually equal to or more than the seed yield remain. Chickpea straw generally contains more protein, greater energy and lower cell wall contents than cereal straws (Kafilzadeh and Maleki, 2011).

Lardy and Anderson (2009) reported that, chickpea straw is higher in nutritive value than cereal straws (44-46% TDN and 4.5-6.5% CP). Chickpea straw can be more palatable than wheat straw, but it is suggested that animals should be allowed to acclimate to the taste before offering large quantities. Bampidis and Christodoulou (2011) concluded that chickpea straw has relatively high metabolisable energy content (7.7 MJ/Kg DM) and can be used as a ruminant feed. Abreu and Bruno-Soares (1998) and Maheri-Sis *et al*. (2011a) suggested that legume straws are usually used in sheep and goat nutrition. Kishore and Sagar (2006) reported that chickpea straw can be used as sole feedstuff for yearling sheep.

Several researchers have reported *in vivo* and *in vitro* organic matter (OM) and crude protein (CP) digestibility of chickpea straw ranged between 47.1-62% and 40-64%, respectively (Lander and Dharmani, 1936; Ramalho Ribeiro and Portugal Melo, 1990; EbnAbbasi *et al.*, 2007; Fekadu *et al.*, 2010; Kafilzadeh and Maleki, 2011). However, there is little information (Ørskov *et al.*, 1992) on *in situ* rumen degradability of chickpea straw. The aim of this experiment was to determine the chemical composition and ruminal OM and CP degradability of chickpea straw using nylon bags (*in situ*) technique.

## Materials and Methods

### Sample Collection and Chemical Analysis

Chickpea straw samples were collected from four local farms in Shabestar, East Azerbaijan province, Iran. Dry matter (DM) determined by drying the samples at 105°C overnight and ash by igniting the samples in muffle furnace at 525°C for 8 h. Ether extract (EE) and crude fiber (CF) content of the samples were determined by soxhlet extraction method and Fiber-Tec system, respectively (AOAC, 1990).

Nitrogen (N) content was measured by the Kjeldahl method and CP was calculated as N*6.25 (AOAC, 1990). Nitrogen free extract (NFE) was calculated using the equation of NFE% = 100 – (CF% + CP% + EE% + Ash %).

### In situ Degradation Procedures

Three ruminally cannulated Ghezel rams (about 55 kg BW) were used to determine *in situ* degradation characteristics. Rams were housed in individual tie stalls bedded with sawdust. Rams fed diets containing alfalfa hay (70%) and concentrate mixture (30%) at the maintenance levels. The concentrate mixture contained 55% barley grain, 15% soybean meal, 5.7% cotton seed meal, 21% wheat bran, 0.3% salt, 1% di calcium phosphate (DCP), 1% calcium carbonate and 1% vitamin- mineral premix.

Dacron bags (18*9 cm; 40-45 micron pore size) were filled with 5 g dried and ground samples then incubated in the rumen of rams for the periods of 0, 2, 4, 8, 12, 24, 48 and 72 h. After the removal of bags from the rumen, bags were rinsed in cold water until water was clear and dried at 60°C for 48 h (Karsli and Russell, 2002; Maheri-Sis *et al.*, 2011b).

Rumen degradation kinetics of OM and CP was fitted by the nonlinear model proposed by Ørskov and McDonald (1979) using FITCURVE software version 6 (Chen, 1995).

P = *a + b* (*1-e^-ct^*)

Where:

P = Percentage of degradability for response variables at t.

*t* = Time relative to incubation (h)

*a* = Highly soluble and readily degradable fraction (%)

*b* = Insoluble and slowly degradable fraction (%)

*c* = Rate constant for degradation (/h)

*e* = 2.7182 (Natural logarithm base)

Following determination of these parameters, the effective degradability of OM and CP in the samples was calculated using and equation described by Ørskov and McDonald (1979):

ED = *a +* (b*c)*/*(*c+k*)

Where:

ED = Effective degradability for response variables (%)

*a* = Highly soluble and readily degradable fraction (%)

*b* = Insoluble and slowly degradable fraction (%)

*c* = Rate constant for degradation (/h)

*k* = Rate constant of passage (/h)

## Results and Discussion

Chemical composition of chickpea straw is presented in Table 1. Most of the obtained values in the current study were in range of previous findings that reported DM, OM, CP, EE and CF content of chickpea straw in ranges of 87-92.2, 86.7-95.3, 3.23-10, 0.5-1.6 and 37-50.6%, respectively (Lander and Dharmani, 1936; Ramalho Ribeiro and Portugal Melo, 1990; Abreu and Bruno-Soares, 1998; Lopez *et al.*, 2005; Lardy and Anderson, 2009; Fekadu *et al.*, 2010; Bampidis and Christodoulou, 2011; Kafilzadeh and Maleki, 2011).

**Table 1:** Chemical composition of chickpea straw on dry matter basis (%).

DM	OM	CP	EE	CF	NFE
92.18	92.00	6.05	5.50	34.30	46.15

Variations in chemical composition of chickpea by-products such as straws can be due to different chickpea varieties, leaf to stem ratio, growing conditions (geographic, seasonal variations, climatic conditions and soil characteristics), extent of foreign materials and impurities such as soil contamination, different measuring methods and laboratories procedures (Ramalho Ribeiro and Portugal Melo, 1990; Maheri-Sis *et al.*, 2007; Bampidis and Christodoulou, 2011; Kafilzadeh and Maleki, 2011).

**Fig. 1:**
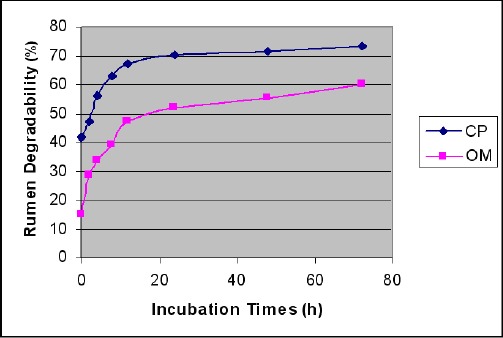
Ruminal organic matter and crude protein degradation of chickpea straw at different incubation times.

Ruminal OM and CP degradation of chickpea straw at different incubation times are illustrated in Table 2 and Figure 1.

**Table 2:** Ruminal organic matter and crude protein degradation of chickpea straw at different incubation times.

Incubation time (h)	Organic matter disappearance (%)	Crude protein disappearance (%)
0	15.08	41.49
2	28.40	47.11
4	33.66	56.24
8	39.33	62.96
12	47.28	67.40
24	52.09	70.16
48	55.16	71.29
72	59.96	73.33

OM and CP degradation at initial incubation times (0-12h) were increased considerably, but degradation rate increments after 12h incubation, were slowl. Final (72h incubation) degradation percentage for OM and CP of chickpea straw were 60.0% and 73.3%, respectively. Ruminal OM and CP degradation characteristics and effective degradability of chickpea straw are shown in Table 3.

**Table 3:** Ruminal organic matter and crude protein degradation parameters and effective degradability of chickpea straw.

Items	Organic matter	Crude protein
*a* (%)	17.50	40.80
*b* (%)	39.20	31.20
*a+b* (%)	56.70	72.00
*c* (/h)	0.116	0.153
ED (%); Out flow rate 0.02 /h	51.00	68.40
ED (%); Out flow rate 0.05 /h	44.90	64.30
ED (%); Out flow rate 0.08 /h	40.70	61.30

*a*, washout fraction as measured by washing loss from nylon bags;

*b*, potentially degradable fraction; *c*, rate of degradation of fraction

*b* (/h). ED: effective degradability

The soluble fraction (*a*), potential degradability (*a+b*) and degradation rate (*c*) of the OM and CP of chickpea straw were 17.5 and 40.8%, 56.7 and 72.0% and 0.116 and 0.153/h, respectively. Effective degradability at different passage rates (2, 5 and 8% per hours) for OM was 51.0, 44.9 and 40.7% and for CP were 68.4, 64.3 and 61.3%, respectively. Lander and Dharmani (1936) illustrated that CP digestibility of chickpea straw was 40% in cattle.

Ramalho Ribeiro and Portugal Melo (1990) reported that OM and CP digestibility of chickpea straw were 62 and 64%, respectively. Abreu and Bruno-Soares (1998) found that OM degradability of chickpea straw was 45.1% and for other legume straws was between 53.9% and 67.7%.

Hadjipanayiotou (2000) reported that OM and CP digestibility of narbon vetch straw was 42.1 and 47.0%. Additionally, CP degradation parameters of narbon vetch straw (i.e. a, b, c and effective degradability) were 13.3%, 23.8%, 10.1%/h and 30.8%, respectively.

EbnAbbasi *et al*. (2007) reported that OM and CP digestibility of chickpea straw were 50.3 and 52.5% respectively. Fekadu *et al*. (2010) using near infrared reflectance spectroscopy (NIRS), found that *in vitro* OM digestibility of chickpea straw ranged from 56.0-58.4%.

Kafilzadeh and Maleki (2011) found that *in vitro* OM digestibility of straw from four varieties of chickpea ranged between 47.1-53.6%. Degradation parameters obtained for chickpea straw in the current study were higher than that of soybean straw reported by Maheri-Sis *et al*. (2011a).

Variation in results between studies may be due to different chemical composition, leaf to stem ratio, method of feedstuff evaluation (*in vivo*, *in vitro* and *in situ*), chickpea varieties, maturity and impurities (Ørskov *et al.*, 1992; Bampidis and Christodoulou, 2011; Kafilzadeh and Maleki, 2011; Maheri-Sis *et al.*, 2011a).

Chumpawadee (2009) and Maheri-Sis *et al*. (2011c) stated that many factors result in variation of *in situ* degradability of feedstuffs, such as chemical composition of samples, bag pore size, sample size, washing procedures, grinding size, diet of experimental animals, species of animal, sample preparation, incubation time and washing method.

## Conclusion

In conclusion, based on chemical composition and degradation characteristics, chickpea straw could have relatively high nutritive value for ruminants. Further investigations are suggested for evaluation of different treatments and supplementation methods to improve nutritional value of chickpea straws.
